# Rural-to-Urban Migrants' Experiences with Primary Care under Different Types of Medical Institutions in Guangzhou, China

**DOI:** 10.1371/journal.pone.0140922

**Published:** 2015-10-16

**Authors:** Jiazhi Zeng, Leiyu Shi, Xia Zou, Wen Chen, Li Ling

**Affiliations:** 1 Faculty of Medical Statistics and Epidemiology, School of Public Health, Sun Yat-sen University, Guangzhou, China; 2 Sun Yat-sen Center for Migrant Health Policy, Sun Yat-sen University, Guangzhou, China; 3 Department of Health Policy and Management, Bloomberg School of Public Health, Johns Hopkins University, Baltimore, United States of America; Örebro University, SWEDEN

## Abstract

**Objectives:**

China is facing the unprecedented challenge of rapidly increasing rural-to-urban migration. Migrants are in a vulnerable state when they attempt to access to primary care services. This study was designed to explore rural-to-urban migrants’ experiences in primary care, comparing their quality of primary care experiences under different types of medical institutions in Guangzhou, China.

**Methods:**

The study employed a cross-sectional survey of 736 rural-to-urban migrants in Guangzhou, China in 2014. A validated Chinese version of Primary Care Assessment Tool—Adult Short Version (PCAT-AS), representing 10 primary care domains was used to collect information on migrants’ quality of primary care experiences. These domains include first contact (utilization), first contact (accessibility), ongoing care, coordination (referrals), coordination (information systems), comprehensiveness (services available), comprehensiveness (services provided), family-centeredness, community orientation and culturally competent. These measures were used to assess the quality of primary care performance as reported from patients’ perspective. Analysis of covariance was conducted for comparison on PCAT scores among migrants accessing primary care in tertiary hospitals, municipal hospitals, community health centers/community health stations, and township health centers/rural health stations. Multiple linear regression models were used to explore factors associated with PCAT total scores.

**Results:**

After adjustments were made, migrants accessing primary care in tertiary hospitals (25.49) reported the highest PCAT total scores, followed by municipal hospitals (25.02), community health centers/community health stations (24.24), and township health centers/rural health stations (24.18). Tertiary hospital users reported significantly better performance in first contact (utilization), first contact (accessibility), coordination (information system), comprehensiveness (service available), and cultural competence. Community health center/community health station users reported significantly better experience in the community orientation domain. Township health center/rural health station users expressed significantly better experience in the ongoing care domain. There were no statistically significant differences across settings in the ongoing care, comprehensiveness (services provided), and family-centeredness domains. Multiple linear regression models showed that factors positively associated with higher PCAT total scores also included insurance covering parts of healthcare payment (P<0.001).

**Conclusions:**

This study highlights the need for improvement in primary care provided by primary care institutions for rural-to-urban migrants. Relevant policies related to medical insurance should be implemented for providing affordable healthcare services for migrants accessing primary care.

## Introduction

China is being challenged by unprecedented large numbers of rural-to-urban migration. Rural-to-urban migrants (245 million) comprised more than one-sixth of the total Chinese populations in 2013 [1]. China’s household registration (hukou) system was implemented in the 1950s, mandating that individuals have household registration for certain rights [2]. Each person was classified as a rural or an urban resident. Since the inception of China’s reform and opening policy, rural workers have migrated to urban areas for better living conditions and employment opportunities. However, the household registration is not easily transferred from rural to urban areas. Rural-to-urban migrants face significant obstacles to access appropriate and timely healthcare [3]. Compared to local residents, the rural-to-urban migrants are rarely entitled to medical insurance. They encounter inequalities when accessing health services and have to pay significantly higher out-of-pocket expenses for healthcare services [4]. In addition, the absence of sick pay and limited spare time may also contribute to poor access to healthcare. Accessible and affordable services have so far failed to be provided to migrants [3].

In 2008, the World Health Organization (WHO) urged that primary care systems be strengthened in all countries and primary care be used as a model to provide fair and efficient care [5]. A large body of research has demonstrated that strong primary care can contribute to better health outcomes, lower health care costs and reducing unnecessary hospital medical care [6–8]. High quality primary care for rural-to-urban migrant populations is important as it has been shown that a strong primary care system can reduce health inequities [9] and mitigate socioeconomic disparities in healthcare utilization [5]. The concepts of primary care have been translated to characteristics that can be measured [10]. They include four core attributes (first contact care, ongoing care, coordinated care and comprehensive care) and three ancillary attributes (family-centered care, community-oriented care and culturally-competent care) [10]. Extensive research has been conducted about the quality of primary care in the USA and Canada.

Over the past two decades, the Chinese government has started to establish a community health service system to provide quality primary care services [11–14]. A key issue of the health care reform plan was expanding and strengthening primary care service institutions to achieve the goal of primary care facilities acting as gatekeepers for hospital services [15]. In an effort to achieve better primary care, the central and local government is investing heavily in primary care service institutions to provide full funding for the staff and zero-profit drugs [16, 17]. Though the government attached great importance to increasing basic medical institutions, primary care services were still underutilized [18]. Patients are still concerned about health service quality in primary care facilities, particularly around general practitioners’ capabilities and the medical resource availability.

The referral system between primary care institutions and municipal hospitals or tertiary hospitals has not yet been completely established in China [19]. Patients have the freedom to select any medical institutions [20]. In China, primary care institutions generally consist of community health centers and community health stations in urban areas, and township health centers and rural health stations in rural areas [16]. In addition, primary care services are also provided by the outpatient department of municipal hospitals and tertiary hospitals [14]. No restriction is made in patients’ freedom to select medical institutions for primary care in terms of insurance policies. Health care expenditures in tertiary hospitals are high and the cost of outpatient visits is growing at a rapid rate. Despite this, patients tend to seek outpatient treatment in tertiary hospitals, even for minor ailments [21, 22]. The concern about general practitioners’ capabilities and medical resource availability was the probable cause. Patients often go directly to higher-tier hospitals, i.e. municipal hospitals and tertiary hospitals, instead of community health centers and community health stations for primary care. However, literature indicated that migrants were more reliant on the primary care system than local residents [23]. Studies showed that migrants were more likely than local residents to use health care in community health centers and community health stations, since the costs would be lower as compared to the costs of using municipal hospitals and tertiary hospitals [24]. In addition, studies have shown that one-quarter of the migrants returned to their household registration place to access primary care [25]. Hence, assessments of migrants’ primary care quality in township health centers and township health stations should be examined. Previous international and internal comparative studies on primary care quality have demonstrated that different models or types of medical institutions could result in different health care performance [26–29]. A better understanding of migrants’ experiences with primary care in the above-mentioned medical institutions is critical for the future development and improvement of primary care services provided to migrants. Until now, this piece of information is lacking.

Underlying the national healthcare system and the migrants’ poor access to primary care services, migrants’ experiences with primary care should be measured, in order to improve the performance of primary care institutions and provide indications of the domains that require further attention. Our study focuses on migrants’ reported access to primary care after migrating to Guangzhou. Migrants’ experiences with primary care in 1) community health centers/community health stations; 2) municipal hospitals and 3) tertiary hospitals in Guangzhou were measured in the survey. For migrants who did not have any experiences utilizing primary care in Guangzhou, their experiences returning to their hometown for primary care in 4) township health centers/township health stations after migrating to Guangzhou were investigated. The goal of the study was to explore rural-to-urban migrants’ experiences with primary care and compare their quality of primary care experiences in above-mentioned four types of medical institutions in Guangzhou, China, in anticipation of providing evidence-based assessment of reform policies. Since healthcare systems are similar across mainland China, this study could provide useful implications for other cities which were experiencing rapid migration growth.

## Materials and Methods

### Study setting

Guangdong is a coastal developed province located in Southern China. With 30% of its total population comprising migrants (31.28 million/104.30 million), it accommodates the largest number of internal migrant population in China [30]. Guangzhou, where this study was conducted, is the capital city of Guangdong province. In Guangzhou, the rural-to-urban migrants make up more than half of its total population (8.37 million/16.69 million) [31].

### Study design

This was a cross-sectional survey conducted in Guangzhou, China, between September and December in 2014. According to the China Migrant Population Dynamic Monitoring Survey 2014, 80.8% of employed migrants work in the following five industries: 1) manufacturing industry; 2) construction industry; 3) wholesale and retail trade; 4) hotels and catering services; and 5) social services [32]. We aimed to recruit 700 participants of which 500 would be recruited from the above-mentioned workplaces and 200 recruited from the communities to ensure that the samples were representative of the migrant population. Considering the estimated loss to follow-up rate of 20%, 875 migrants were needed for study inclusion.

The comparison of the quality of primary care experiences between community health center/community health station users and tertiary hospital users was a primary objective for the current study. Based on findings from a previous paper [33], we estimated that the difference between the means of PCAT total scores between these two types of medical institutions was 2.2 and the pooled standard deviation was 5.5. Based on the sample size calculation, [$N=\frac{4{\left(t_{\alpha /2}+t_{\beta }\right)}^{2}S^{2}}{\delta^{2}}$, *n*_1_ = *n*_2_ = N/2, where *N* was for total sample size of two groups, *n*_1_ and *n*_2_ were for the sample size of community health center/community health station users and tertiary hospital users, respectively, *α* was set at 0.05, *β* was set at 0.1, *S* was for pooled standard deviation of PCAT scores, *δ* was for difference between the means of PCAT scores], a minimum sample size of 132 participants accessing primary care in community health centers/community health stations and tertiary hospitals, respectively, were needed to provide the required power (1-*β* = 0.9) for comparing the PCAT total scores between community health center/community health station users and tertiary hospital users. Participants were recruited from workplaces and communities, thus the type of medical institution they accessed for primary care was unknown before the survey. We aimed to recruit 700 participants to meet the minimum sample size of 132 community health center/community health station users and tertiary hospital users, respectively.

A multistage sampling method was used. In stage one, with the help of the local government officials and the community residential committees, a list of the workplaces and communities with high concentrations of migrants was compiled. Workplaces and communities which provided consent to the study were coded. The researcher used computer-generated random numbers to choose 5 manufacturing factories, 5 construction sites, 50 self-employed ventures, 10 restaurants, 5 social services working places, and 10 communities using the simple random sampling selection method. In stage two, we aimed to approach 25 participants from each manufacturing factory, 25 from each construction site, 2 or 3 from each self-employed venture, 12 or 13 from each restaurant, 25 from each social service work place, and 25 from each community. In total, we aimed to approach 125 participants from each of the above-mentioned workplaces and 250 participants from the communities. We recruited subjects who: 1) were aged 18 and above; 2) held rural household registration outside Guangzhou; 3) used community health centers, community health stations, municipal hospitals or tertiary hospitals in Guangzhou or township health centers or rural health stations in their hometown after migrating to Guangzhou; and 4) provided informed consent.

In all, a total of 850 migrants were approached for study inclusion (some workplaces had fewer persons who met the criteria), 736 consented to participate in the survey (response rate of 86.6%), and 114 refused to participate. The most common reason for non-response was having no time. The language matter was not the cause of non-response, all migrants approached could speak mandarin, the official language of China. 521 participants were recruited from workplaces (response rate between 83.1% and 90.1%). 215 participants were recruited from communities (response rate of 86.0%), among them, 83 had a job working at the above-mentioned workplaces, 23 had other jobs, and 109 were unemployed. In total, 115 (15.6%) participants were manufacturing industry workers, 110 (14.9%) were construction industry workers, 121 (16.4%) worked on wholesale and retail trade, 128 (17.4%) worked on hotels and catering services, 130 (17.7%) worked on social services, 23 (3.2%) had other jobs, and 109 (14.8%) were unemployed. The description of the sampling results is presented in Table 1.

**Table 1:** Description of the sampling results.

Occupation	Recruited from workplaces		Recruited from communities		Total n(%)
	Participants n	Response rate %	Participants n	Response rate %	
Manufacturing industry	100	84.0	15	-	115(15.6)
Construction industry	98	83.1	12	-	110(14.9)
Wholesale and retail trade	105	88.2	16	-	121(16.4)
Hotels and catering services	109	88.6	19	-	128(17.4)
Social services	109	90.1	21	-	130(17.7)
Other jobs	-	-	23	-	23(3.2)
Unemployed	-	-	109	-	109(14.8)
Total	521	86.8	215	86.0	736(100.0)

### Data collection

The Chinese version of Primary Care Assessment Tool—Adult Short Version (PCAT-AS) was used for data collection. The PCAT was developed by Starfield and Shi at the Johns Hopkins Primary Care Policy Center [8, 26]. Based on the concepts outlined by the WHO and Institute of Medicine definitions [34, 35], the PCAT-AS assesses patients’ quality of primary care experiences rather than their satisfaction [36]. Studies have shown that the PCAT has satisfactory validity and reliability [26]. The PCAT-AS has been translated into Cantonese Chinese from English. The Chinese version of PCAT-AS has good validity and reliability [20, 33, 37]. The PCAT-AS consists of 10 domains [38]. First contact (utilization) measures whether the primary care institutions act as gatekeepers while first contact (accessibility) reflects whether patients are able to access primary care when a new medical need or health problem arises. Ongoing care refers to the longitudinal use of a regular source of care and the relationship between the provider of source of care and the patient. Coordination (referrals) measures the linking of health care visits and services between different levels of medical institutions while coordination (information systems) measures whether the electronic information systems access medical records for patients. Comprehensiveness (services available) refers to the availability of the services in primary care while comprehensiveness (services provided) refers to the appropriate provision of primary care services. Family centeredness reflects the participation of the family members in the treatment of a patient. Community orientation refers to the provider’s knowledge of community health care needs. Cultural competence refers to whether the patient recommends the primary care provider to others [38]. Specific PCAT items are included in the S1 Appendix. Answers to the PACT-AS are ranked on a 4-point Likert-type scale with “1” for “Definitely not”, “2” for “Probably not”, “3” for “Probably”, and“4” for “Definitely”, while “9” for “Not sure/Do not remember”.

The survey also covered information including socio-demographic characteristics (i.e., age, gender, marital status, education, employment status, household-income), migrant characteristics (i.e., permanent migration intention, migration with family, time of migration, years of residence in Guangzhou), health status (i.e., self-rated health status, chronic disease status), health insurance status (i.e., present health insurance status, source of health payment) and health care service utilization (i.e., primarily medical institution for primary care). The question “Is there a doctor or place that you usually go if you are sick or need advice about your health?” was used to define the medical institution for primary care. The survey was conducted at the migrants’ residence or workplace through face-to-face interviews. Migrants recruited from the communities were interviewed at their residence. It took about 25 minutes to finish the survey. Small household product gifts (worth about RMB 20, or USD 3.22) were given to the survey participants as reward upon their completion of the survey. 14 postgraduate students from School of Public Health at Sun Yat-Sen University were trained to conduct the survey. They received the training for two days from the supervisor. All the completed questionnaires were checked for completeness and consistency.

### Data analysis

Data were double-entered into the Epidata 3.1 before being exported to SPSS (USA, 20.0) for analysis. PCAT scores were assessed for item scores, domain scores and total scores. The domain scores were calculated by the sum scores of the items divided by the number of items to produce mean scores. The total scores were calculated by summing the mean scores for the 9 domains except the coordination (referrals) domain since only 60 participants reported having an experience of referral. The “Not sure/Do not remember” answers were scored as “2” for all domains, except comprehensiveness (services provided), where the “Not sure/Do not remember” answers were converted to “0”, according to the scoring instructions provided in the PCAT Manual [38].

Descriptive statistics presented means and standard errors for continuous variables, while proportions and rates were calculated for categorical variables. Chi-square test was used to test for differences of characteristics among the migrants accessing different types of medical institutions. T-test was used for comparison on domain scores and total scores by variables. Analysis of variance (ANOVA) and analysis of covariance (ANCOVA) were conducted for comparison on domain scores and total scores among the migrants accessing different types of medical institutions. Differences in the means of adjusted scores between every two medical institutions were also compared using the Bonferroni *t*-test. Four multiple linear regression models were used to explore factors associated with PACT total scores: model I including only medical institution type, model II to IV controlling for the characteristics (i.e., socio-demographic characteristics, migrant characteristics, health status and health insurance status). A p-value<0.05 was considered statistically significant in the analysis.

### Ethics

The study was approved by the Institutional Review Board (IRB) of the School of Public Health, Sun Yat-sen University, China. Written consent was sought from eligible participants. All the participants were given a description of the study and assured of keeping their identity and responses confidential before the survey. Names were not written on the questionnaire. Participants could leave their phone numbers on the questionnaire if they agreed to participate in the follow-up study. Giving small household product gifts to the survey participants was ethically allowed since the University IRB has approved this. Hard copies of the questionnaires were put in a secured room. Only the supervisor and the data administrator had the password to open the secured soft copies of the questionnaires.

## Results

Table 2 summarizes the characteristics of the participants. Migrants accessed primary care in four types of institutions, and differed by age, marital status, permanent migration intention, migration with family, years of residence in Guangzhou, chronic disease status, and source of health payment. Township health center/rural health station users were more likely to be aged 18~59, not to have permanent migration intention, live in Guangzhou for less than 5 years and not to have any chronic disease. Community health center/community health station users were more likely to have out-of-pocket payment for health care. Tertiary hospital users were more likely to be married and migrate with family.

**Table 2:** Comparison of characteristics among migrants accessing primary care in four types of medical institutions.

Characteristics	All participants (%) n = 736(100.0)	THC/RHS (%) n = 181(24.6)	CHC/CHS (%) n = 215(29.2)	Municipal hospital (%) n = 128(17.4)	Tertiary hospital (%) n = 212(28.8)	*χ* ^*2*^	P
**Socio-demographic characteristics**							
Age (years)						9.43	0.024
18~59	659 (89.5)	170(93.9)	182(84.7)	116(90.6)	191(90.1)		
≥60	77(10.5)	11(6.1)	33(15.3)	12(9.4)	21(9.9)		
Gender						0.44	0.932
Male	386(52.4)	92(50.8)	116(54.1)	66(51.4)	112(53.0)		
Female	350(47.6)	89(49.2)	99(45.9)	62(48.6)	100(47.0)		
Marital status						10.55	0.014
Unmarried	207(28.1)	63(34.8)	66(30.7)	34(26.6)	44(20.8)		
Married	529(71.9)	118(65.2)	149(69.3)	94(73.4)	168(79.2)		
Level of education						5.72	0.126
Junior high school or below	375(51.0)	102(56.4)	96(44.7)	66(51.6)	111(52.4)		
Senior high school or above	361(49.0)	79(43.6)	119(55.3)	62(48.4)	101(47.6)		
Employment status						6.84	0.077
Unemployed	109(14.8)	29(16.0)	41(19.1)	17(13.6)	22(10.2)		
Employed	627(85.2)	152(84.0)	174(80.9)	111(86.4)	190(89.8)		
Monthly household income per capita (RMB)						6.72	0.081
<3000	399(54.2)	113(62.4)	109(50.7)	68(53.1)	109(51.4)		
≥3000	337(45.8)	68(37.6)	106(49.3)	60(46.9)	103(48.6)		
**Migrant characteristics**							
Permanent migration intention						10.73	0.013
No	592(80.4)	155(85.6)	159(74.0)	109(85.2)	169(79.7)		
Yes	144(19.6)	26(14.4)	56(26.0)	19(14.8)	43(20.3)		
Migration with family						12.70	0.005
No	230(31.3)	74(40.9)	57(26.5)	43(33.6)	56(26.4)		
Yes	506(68.7)	107(59.1)	158(73.5)	85(66.4)	156(73.6)		
Times of migration						5.21	0.157
1	356(48.4)	81(44.8)	96(44.7)	64(50.0)	115(54.2)		
≥2	380(51.6)	100(55.2)	119(55.3)	64(50.0)	97(45.8)		
Years of residence in Guangzhou						22.09	<0.001
≤5	400(54.3)	116(64.1)	123(57.2)	73(57.0)	88(41.5)		
>5	336(45.7)	65(35.9)	92(42.8)	55(43.0)	124(58.5)		
**Health status**							
Self-rated health status						1.89	0.595
Fair/Poor/Very poor	236(32.1)	51(28.2)	74(34.4)	41(32.0)	70(33.0)		
Excellent/Good	500(67.9)	130(71.8)	141(65.6)	87(68.0)	142(67.0)		
Chronic disease status						8.10	0.044
No chronic disease	642(87.2)	165(91.2)	192(89.3)	104(81.3)	181(85.4)		
Any chronic disease	94(12.8)	16(8.8)	23(10.7)	24(18.7)	31(14.6)		
**Health insurance status**							
Present of health insurance						3.02	0.388
No	109(14.8)	27(14.9)	28(13.0)	25(19.5)	29(13.7)		
Yes	627(85.2)	154(85.1)	187(87.0)	103(80.5)	183(86.3)		
Source of health payment						28.77	<0.001
Out of pocket only	469(63.7)	87(48.1)	158(73.5)	85(66.4)	139(65.6)		
Insurance covering parts	267(36.3)	94(51.9)	57(26.5)	43(33.6)	73(34.4)		

Note: THC = Township health center; RHS = Rural health station; CHC = Community health center; CHS = Community health station.

1RMB = 0.16USD.

Table 3 presents the results of the comparison of PCAT total scores and domain scores among migrants with different characteristics. Migrants who were aged ≥60, unemployed, migrated with family, migrated once, had health insurance, and had insurance covering parts of health payment reported higher PCAT total scores. Significant age, gender, marital status, employment status, permanent migration intention, migration with family, migration times, years of residence in Guangzhou, self-rated health status, chronic disease status, and source of health payment differences in the PCAT domain scores were noted. Specifically, migrants who had insurance covering parts of health payment reported better quality of primary care experiences of first contact-utilization, first contact-accessibility, ongoing care, coordination care-referrals, comprehensive care-services available and family-centered care.

**Table 3:** Comparison of primary care assessment scores among migrants with different characteristics.

Variables	First contact (Utilization) Mean (SE)	First contact (Accessibility) Mean (SE)	Ongoing care Mean (SE)	Coordination (Referrals) Mean (SE)	Coordination (Information systems) Mean (SE)	Comprehensiveness (Services available) Mean (SE)	Comprehensiveness (Services provided) Mean (SE)	Family centeredness Mean (SE)	Community orientation Mean (SE)	Culturally competent Mean (SE)	Total scores Mean (SE)
**Socio-demographic characteristics**											
Age (years)											
18~59	2.79(0.03)	2.87(0.03)	2.24(0.03)	2.71(0.11)	3.21(0.04)	3.09(0.03)	2.62(0.03)	3.00(0.03)	1.81(0.03)	2.96(0.03)	24.61(0.15)
≥60	2.84(0.07)	2.80(0.06)	2.37(0.06)	2.88(1.13)	3.48(0.08)**	3.12(0.08)	2.82(0.08)*	2.91(0.09)	2.11(0.07)***	3.06(0.06)	25.51(0.41)*
Gender											
Male	2.82(0.05)	2.89(0.04)	2.27(0.04)	2.67(0.19)	3.07(0.05)	3.06(0.05)	2.70(0.05)	2.98(0.05)	1.85(0.05)	2.92(0.04)	24.56(0.23)
Female	2.78(0.04)	2.84(0.03)	2.25(0.03)	2.75(0.14)	3.36(0.04)***	3.12(0.04)	2.61(0.04)	3.00(0.04)	1.85(0.04)	3.00(0.03)	24.83(0.18)
Marital status											
Unmarried	2.77(0.06)	2.85(0.05)	2.26(0.05)	2.67(0.20)	3.00(0.07)	3.08(0.06)	2.73(0.05)	2.96(0.06)	1.84(0.06)	2.90(0.05)	24.37(0.27)
Married	2.81(0.03)	2.87(0.03)	2.26(0.03)	2.74(0.13)	3.34(0.04)***	3.1(0.04)	2.62(0.03)	3.01(0.04)	1.85(0.03)	3.00(0.03)	24.86(0.17)
Level of education											
Junior high school or below	2.78(0.04)	2.89(0.04)	2.26(0.03)	2.85(0.16)	3.28(0.05)	3.12(0.04)	2.65(0.04)	2.98(0.05)	1.83(0.04)	3.00(0.04)	24.80(0.21)
Senior high school or above	2.82(0.04)	2.84(0.03)	2.26(0.04)	2.58(0.15)	3.21(0.05)	3.07(0.04)	2.64(0.04)	3.00(0.04)	1.87(0.04)	2.94(0.03)	24.64(0.20)
Employment status											
Unemployed	2.84(0.05)	2.81(0.04)	2.30(0.04)	2.73(0.21)	3.40(0.05)	3.10(0.05)	2.73(0.05)	3.00(0.06)	2.00(0.05)	3.00(0.04)	25.19(0.26)
Employed	2.78(0.04)	2.89(0.03)	2.24(0.03)	2.71(0.13)	3.18(0.04)**	3.09(0.04)	2.61(0.04)*	2.99(0.04)	1.79(0.03)***	2.95(0.03)	24.52(0.17)*
Monthly household income per capita (RMB)											
<3000	2.80(0.04)	2.87(0.03)	2.25(0.03)	2.50(0.15)	3.22(0.05)	3.11(0.04)	2.63(0.04)	2.94(0.04)	1.84(0.04)	2.97(0.03)	24.63(0.19)
≥3000	2.81(0.04)	2.86(0.04)	2.26(0.04)	2.89(0.15)	3.28(0.05)	3.08(0.05)	2.67(0.04)	3.06(0.05)	1.86(0.04)	2.96(0.04)	24.83(0.22)
**Migrant characteristics**											
Permanent migration intention											
No	2.79(0.03)	2.86(0.03)	2.24(0.03)	2.64(0.12)	3.21(0.04)	3.09(0.03)	2.65(0.03)	3.00(0.04)	1.84(0.03)	2.97(0.03)	24.64(0.16)
Yes	2.83(0.06)	2.88(0.05)	2.34(0.06)	3.14(0.22)	3.41(0.07)*	3.13(0.07)	2.62(0.07)	2.97(0.07)	1.87(0.06)	2.99(0.06)	25.03(0.33)
Migration with family											
No	2.79(0.05)	2.87(0.05)	2.22(0.05)	2.52(0.21)	2.96(0.07)	3.07(0.05)	2.66(0.06)	2.97(0.06)	1.82(0.05)	2.89(0.05)	24.25(0.26)
Yes	2.81(0.03)	2.86(0.03)	2.27(0.03)	2.79(0.13)	3.37(0.04)***	3.10(0.04)	2.64(0.03)	3.00(0.04)	1.87(0.03)	3.00(0.03)*	24.93(0.17)*
Times of migration											
1	2.85(0.04)	2.91(0.04)	2.33(0.04)	2.63(0.18)	3.28(0.05)	3.14(0.04)	2.68(0.04)	2.98(0.05)	1.86(0.04)	3.01(0.04)	25.03(0.21)
≥2	2.75(0.04)	2.82(0.03)	2.19(0.04)**	2.78(0.13)	3.21(0.05)	3.06(0.04)	2.62(0.04)	3.01(0.04)	1.84(0.04)	2.93(0.03)	24.43(0.20)*
Years of residence in Guangzhou											
≤5	2.79(0.04)	2.87(0.03)	2.25(0.03)	2.73(0.17)	3.14(0.05)	3.13(0.04)	2.72(0.04)	3.02(0.04)	1.87(0.04)	2.95(0.03)	24.73(0.19)
>5	2.81(0.04)	2.86(0.04)	2.27(0.04)	2.71(0.14)	3.37(0.05)***	3.06(0.05)	2.56(0.04)**	2.95(0.05)	1.83(0.04)	2.99(0.04)	24.70(0.22)
**Health status**											
Self-rated health status											
Fair/Poor/Very poor	2.80(0.05)	2.80(0.04)	2.27(0.04)	2.57(0.17)	3.34(0.06)	2.96(0.06)	2.54(0.05)	2.95(0.05)	1.84(0.04)	2.93(0.04)	24.43(0.24)
Excellent/Good	2.80(0.04)	2.90(0.03)	2.25(0.03)	2.82(0.15)	3.20(0.04)*	3.16(0.04)**	2.70(0.04)**	3.01(0.04)	1.85(0.04)	2.99(0.03)	24.86(0.18)
Chronic disease status											
No chronic disease	2.79(0.03)	2.87(0.03)	2.24(0.03)	2.70(0.12)	3.23(0.04)	3.13(0.03)	2.66(0.03)	3.02(0.03)	1.84(0.03)	2.96(0.03)	24.74(0.15)
Any chronic disease	2.87(0.08)	2.85(0.07)	2.39(0.07)*	2.83(0.35)	3.35(0.08)	2.85(0.09)**	2.53(0.08)	2.81(0.09)*	1.89(0.07)	3.04(0.06)	24.59(0.40)
**Health insurance status**											
Present of health insurance											
No	2.82(0.08)	2.75(0.06)	2.26(0.07)	2.69(0.23)	3.12(0.09)	2.99(0.08)	2.54(0.07)	2.91(0.08)	1.76(0.07)	2.87(0.07)	24.03(0.35)
Yes	2.80(0.03)	2.88(0.03)	2.26(0.03)	2.72(0.13)	3.27(0.04)	3.11(0.03)	2.66(0.03)	3.00(0.03)	1.86(0.03)	2.99(0.03)	24.84(0.16)*
Source of health payment											
Out of pocket only	2.75(0.04)	2.78(0.03)	2.21(0.03)	2.51(0.12)	3.21(0.04)	3.02(0.04)	2.61(0.04)	2.93(0.04)	1.83(0.04)	2.97(0.03)	24.32(0.18)
Insurance covering parts	2.89(0.05)*	3.01(0.04)***	2.34(0.05)*	3.11(0.20)**	3.30(0.06)	3.23(0.05)**	2.71(0.05)	3.09(0.05)*	1.89(0.05)	2.96(0.04)	25.43(0.23)***

Note: Only 60 migrants reported having an experience of referral, the total scores were calculated by summing the mean scores for 9 domains except the Coordination (Referrals) domain.

T-test was used to compare PACT domain scores and total scores by variables.

***P<0.001

**P <0.01

*P<0.05

1RMB = 0.16USD.

After adjustments were made, means of PCAT total scores were 24.86 among the participants, the coordination (information systems) domain score was the highest (3.25) and community orientation domain score was the lowest (1.84) among all domains. Migrants accessing primary care in tertiary hospitals (25.49) reported the highest PCAT total scores, followed by municipal hospitals (25.02), community health centers/community health stations (24.24), and township health centers/rural health stations (24.18). Tertiary hospital users reported significantly better performance in first contact (utilization), first contact (accessibility), coordination (information system), comprehensiveness (service available), and cultural competence. Community health center/community health station users reported significantly better experience in the community orientation domain. Township health center/rural health station users expressed significantly better experience in the ongoing care domain. There were no statistically significant differences across settings in the ongoing care, comprehensiveness (services provided), and family-centeredness domains (Table 4).

**Table 4:** Comparison of primary care assessment scores among migrants accessing primary care in four types of medical institutions.

Primary care domains	All participants Mean (SE)	THC/RHS Mean (SE)	CHC/CHS Mean (SE)	Municipal hospital Mean (SE)	Tertiary hospital Mean (SE)	*F*	P
**Unadjusted**							
First contact (Utilization)	2.80(0.03)	2.68(0.06)	2.56(0.05)	2.90(0.06)	3.08(0.05)	19.55	<0.001
First contact (Accessibility)	2.86(0.02)	2.96(0.05)	2.66(0.04)	2.84(0.06)	3.00(0.05)	11.12	<0.001
Ongoing care	2.26(0.03)	2.38(0.05)	2.20(0.05)	2.28(0.06)	2.21(0.05)	2.79	0.040
Coordination (Referrals)	2.72(0.11)	2.93(0.23)	2.61(0.18)	2.54(0.32)	2.82(0.20)	0.51	0.675
Coordination (Information systems)	3.25(0.03)	2.86(0.08)	3.15(0.06)	3.38(0.07)	3.59(0.05)	25.12	<0.001
Comprehensiveness (Services available)	3.10(0.03)	3.07(0.06)	3.00(0.05)	3.05(0.07)	3.24(0.06)	3.53	0.015
Comprehensiveness (Services provided)	2.65(0.03)	2.68(0.06)	2.67(0.05)	2.73(0.07)	2.54(0.06)	1.91	0.127
Family centeredness	2.99(0.03)	2.98(0.07)	2.98(0.06)	3.07(0.07)	2.97(0.06)	0.38	0.768
Community orientation	1.85(0.03)	1.88(0.06)	1.99(0.05)	1.81(0.06)	1.70(0.05)	5.48	0.001
Culturally competent	2.97(0.02)	2.79(0.05)	3.02(0.04)	2.88(0.06)	3.12(0.05)	9.29	<0.001
Total scores	24.72(0.14)	24.26(0.29)	24.24(0.26)	24.94(0.32)	25.46(0.27)	4.73	0.003
**Adjusted**							
First contact (Utilization)	2.81(0.03)	2.66(0.06)^b^ ^,^ ^c^	2.57(0.05)^d^ ^,^ ^e^	2.90(0.07)^b^ ^,^ ^d^	3.09(0.05)^c^ ^,^ ^e^	19.27	<0.001
First contact (Accessibility)	2.90(0.03)	2.91(0.05)^a^	2.69(0.05)^a^ ^,^ ^e^	2.85(0.06)	3.01(0.05)^e^	8.68	<0.001
Ongoing care	2.28(0.03)	2.38(0.05)^a^ ^,^ ^c^	2.20(0.05)^a^	2.27(0.06)	2.20(0.05)^c^	2.79	0.039
Coordination (Referrals)	2.91(0.14)	2.51(0.32)	2.60(0.19)	2.60(0.38)	3.05(0.22)	0.97	0.415
Coordination (Information systems)	3.25(0.04)	2.90(0.07)^a^ ^,^ ^b^ ^,^ ^c^	3.14(0.06)^a^ ^,^ ^d^ ^,^ ^e^	3.40(0.08)^b^ ^,^ ^d^	3.55(0.06)^c^ ^,^ ^e^	20.97	<0.001
Comprehensiveness (Services available)	3.15(0.04)	3.02(0.06)^c^	3.00(0.06)^e^	3.08(0.07)	3.26(0.06)^c^ ^,^ ^e^	4.43	0.004
Comprehensiveness (Services provided)	2.66(0.03)	2.66(0.06)	2.65(0.05)	2.75(0.07)	2.57(0.05)	1.34	0.259
Family centeredness	3.00(0.04)	2.96(0.07)	2.98(0.06)	3.08(0.08)	2.98(0.06)	0.53	0.663
Community orientation	1.84(0.03)	1.90(0.06)	1.97(0.05)^e^	1.81(0.07)	1.71(0.05)^e^	4.69	0.003
Culturally competent	2.97(0.03)	2.79(0.05)^a^ ^,^ ^c^	3.03(0.05)^a^	2.88(0.06)^f^	3.12(0.05)^c^ ^,^ ^f^	8.42	<0.001
Total scores	24.86(0.16)	24.18(0.29)^c^	24.24(0.27)^e^	25.02(0.34)	25.49(0.26)^c^ ^,^ ^e^	5.23	0.001

Note: THC = Township health center; RHS = Rural health station; CHC = Community health center; CHS = Community health station.

Only 60 migrants reported having an experience of referral, the total scores were calculated by summing the mean scores for 9 domains except the Coordination (Referrals) domain.

ANOVA carried out for unadjusted domain scores and ANCOVA carried out for adjusted domain scores, which were adjusted for age, gender, marital status, level of education, employment status, monthly household income per capita, permanent migration intention, migration with family, times of migration, years of residence in Guangzhou, self-rated health status, chronic disease status, present of health insurance and source of health payment.

Bonferroni t-test had P<0.008:

^a^ THC/RHS vs. CHC/CHS

^b^ THC/RHS vs. MH

^c^ THC/RHS vs. TH

^d^ CHC/CHS vs. MH

^e^ CHC/CHS vs. TH

^f^ MH vs.TH

The linear regression models show that the medical institutions type and source of health payment were significantly associated with the PCAT total scores. In model I-IV, after controlling for various characteristics of the migrants, migrants who described township health centers/rural health stations and community health centers/community health stations as their main primary care providers had significantly lower PCAT total scores when compared with those who accessed primary care through tertiary hospitals. There was no statistical significant difference for PCAT total scores between tertiary hospital users and municipal hospital users. The results of these models also showed that socio-demographic characteristics, migrant characteristics, health status and presence of health insurance were not associated with the PCAT total scores. Specifically, in model IV, insurance covering parts of healthcare payment was significantly associated with higher PCAT total scores (P<0.001) (Table 5).

**Table 5:** Linear regression analysis on primary care assessment total scores.

Variables	Model I		Model II		Model III		Model IV	
	*β* (95% CI)	P	*β* (95% CI)	P	*β* (95% CI)	P	*β* (95% CI)	P
**Medical institution type**								
Tertiary hospital (ref)								
THC/RHS	-1.20(-1.96,-0.44)	0.002	-1.03(-1.81,-0.25)	0.009	-1.08(-1.86,-0.29)	0.007	-1.31(-2.09,-0.52)	0.001
CHC/CHS	-1.22(-1.95,-0.49)	0.001	-1.25(-1.99,-0.52)	0.001	-1.28(-2.02,-0.54)	0.001	-1.25(-1.98,-0.51)	0.001
Municipal hospital	-0.52(-1.36,0.32)	0.227	-0.51(-1.35,0.34)	0.239	-0.52(-1.36,0.33)	0.234	-0.47(-1.31,0.37)	0.272
**Socio-demographic characteristics**								
Age (years)								
18~59 (ref)								
>60			0.59(-0.44,1.61)	0.261	0.49(-0.55,1.52)	0.354	0.72(-0.34,1.78)	0.182
Gender								
Male (ref)								
Female			0.06(-0.54,0.66)	0.844	-0.03(-0.64,0.58)	0.925	0.06(-0.55,0.66)	0.856
Marital status								
Unmarried (ref)								
Married			0.24(-0.42,0.89)	0.479	0.17(-0.56,0.91)	0.643	-0.08(-0.82,0.66)	0.829
Level of education								
Junior high school or below (ref)								
Senior high school or above			-0.06(-0.64,0.51)	0.834	-0.13(-0.72,0.45)	0.657	-0.23(-0.81,0.36)	0.448
Employment status								
Unemployed (ref)								
Employed			-0.40(-1.17,0.37)	0.309	-0.14(-0.94,0.67)	0.735	-0.34(-1.14,0.46)	0.405
Monthly household income per capita (RMB)								
<3000 (ref)								
≥3000			0.26(-0.32,0.83)	0.381	0.34(-0.25,0.92)	0.257	0.26(-0.32,0.83)	0.385
**Migrant characteristics**								
Permanent migration intention								
No (ref)								
Yes					0.26(-0.49,1.01)	0.493	0.28(-0.47,1.02)	0.467
Migration with family								
No (ref)								
Yes					0.48(-0.27,1.24)	0.205	0.47(-0.28,1.21)	0.220
Times of migration								
1 (ref)								
≥2					-0.42(-1.00,0.17)	0.164	-0.36(-0.94,0.22)	0.222
Years of residence in Guangzhou								
≤5 (ref)								
>5					-0.47(-1.09,0.15)	0.134	-0.41(-1.02,0.21)	0.196
**Health status**								
Self-rated health status								
Fair/Poor/Very poor (ref)								
Excellent/Good							0.46(-0.17,1.08)	0.150
Chronic disease status								
No chronic disease (ref)								
Any chronic disease							-0.54(-1.44,0.35)	0.234
**Health insurance status**								
Present of health insurance								
No (ref)								
Yes							0.41(-0.39,1.21)	0.312
Source of health payment								
Out of pocket only (ref)								
Insurance covering parts							1.15(0.55,1.75)	<0.001

Note: Model I: Included only medical institution type. Model II: Controlled for socio-demographic characteristics. Model III: Controlled for socio-demographic characteristics, migrant characteristics. Model IV: Controlled for socio-demographic characteristics, migrant characteristics, health status and health insurance status.

THC = Township health center; RHS = Rural health station; CHC = Community health center; CHS = Community health station.

1RMB = 0.16USD.

## Discussion

This study is the first to evaluate the relationship between rural-to-urban migrants’ experiences in primary care, given varied types of medical institution use, using an internationally developed and validated tool in China. Our results add to the evidence indicating that the quality of primary care measured by rural-to-urban migrants’ experience is related to the type of medical institutions they use for care. After adjusting for socio-demographic characteristics, migrant characteristics, health status and health insurance status, migrants at tertiary hospitals reported the highest PCAT total scores, followed by municipal hospitals and then community health centers and community health stations, township health centers and rural health stations, with the lowest scores. Though information of characteristics of migrants who refused to be interviewed was absent, there were no statistically significant differences between the migrant population of Guangzhou and the participants of our survey with respect to age and gender. However, the participants of our survey had higher education level than the migrant population of Guangzhou [31].

Previous studies have used PCAT to assess primary care and explored the relationship between patients’ experiences with primary care and types of providers [14, 26, 27, 29]. The community health centers in the USA are non-profit medical institutions and users of US community health centers have characteristics associated with poor care, whereas, the PCAT scores of US community health center users were evaluated to be higher [26]. The results of our study show inconsistency with a previous study conducted in China which found that community health centers and community health stations outperformed hospitals among non-migrant users [14]. This was an unexpected finding in the context of heavy capital investments being made for primary care institutions to achieve better primary care. Research suggested that migrants were more likely to seek healthcare from community health centers due to the lower costs as compared to hospitals [23]. Nonetheless, in our study migrants did not give community health centers and community health stations the highest PCAT total scores.

After adjusting for potential confounders, the results indicated that migrants accessing primary care in tertiary hospitals reported the highest first contact (utilization and accessibility), coordination (information system), comprehensiveness (service available) and culturally competent domain scores. Migrants accessing primary care in community health centers and community health stations did not show superior scores in any domains except community orientation. Those accessing primary care in township health centers and rural health stations were likely to express the highest ongoing care domain scores.

The highest scores for first-contact care (utilization and accessibility) for tertiary hospitals suggested the goal of primary care institutions acting as gatekeepers for hospital services had not been achieved. With the absence of referral system between primary care institutions and hospitals, migrants have freedom for doctor shopping [20]. Our study showed that migrants tended to go for primary care in tertiary hospitals, and this finding was in line with previous studies [21, 22]. However, a recent study conducted in Shenzhen, a city which also accommodates large number of migrants from Guangdong province, showed that migrants covered by Medical Insurance System for Migrant Employees (MISM) would visit primary care institutions for first-contact and obtain referrals to hospitals due to the health insurance reimbursement stipulation [23]. An effective implementation of the preferential policy, which stipulates that migrants could be reimbursed for healthcare expenditures occurring at the community health centers and community health stations, may help migrants choose these primary care institutions for first-contact and reduce the burden on the medical expenses.

The highest scores of township health centers and rural health stations in the ongoing care domain gave the evidence that migrants considered doctors in these primary care institutions in their hometown to be more familiar with their health condition. Ongoing care over time is intended to help the provider and the patient build a long-term relationship to foster mutual understanding between provider and patient and knowledge of both as to the other’s expectations and needs [38]. Previous studies found that smaller practices had higher continuity. In larger practices, physicians had more colleagues to rely on to cover their patients [39]. Township health centers and rural health stations in rural areas are smaller medical institutions compared to community health centers and community health stations in urban areas [40]. This could improve the relational continuity between providers and patients and increase the providers’ knowledge of the patients’ needs. In addition, low wages for doctors and limited opportunity for career progression in community health centers and community health stations resulted in frequent staff changes, and might further affect continuity of care [14]. Residential mobility and frequent workplace changes presumably weakened continuity of care, resulting in doctors’ lack of knowledge about migrants’ medical history and important health problems. Our study corroborates perceptions that there may be barriers for migrants to achieve ongoing care in the relocated city.

Of all the participants, only 60 migrants reported experiencing a referral. There was no significant difference in the coordination (referrals) domain scores among migrants accessing primary care in four types of medical institutions. Therefore, the PCAT total scores were calculated by summing the mean scores for the 9 domains except the coordination (referrals) domain. As discussed in the introduction section, the limited referral system resulted in free choice for patients to decide where to seek primary care, and thus weaken the appropriate referrals [41]. There was a strong consensus among health policy experts that a large proportion of patients who went directly to hospital could have also been treated properly in primary care institutions [42]. The establishment of a two-way referral system that encourages an exchange of patients between primary care institutions and hospitals may require government and insurance regulations. The adjusted coordination (information system) domain scores were the highest for any domains because tertiary hospitals, municipal hospitals, community health centers and community health stations had adopted electronic medical records. The coordination function is important as availability of medical information and prevalence of chronic diseases are increasing.

A significant difference exists between adjusted mean scores in the comprehensiveness (service available) domain, with tertiary hospitals scoring highest, and community health centers and community health stations scoring lowest. Comprehensiveness (services available) measures the availability of the services in primary care, including family planning services, mental health problems counseling, wound suture services and immunizations. It has been shown that low reproductive health knowledge level, high psychological distress, and high incidence of workplace injury are emerging public health priorities among migrants in China [43–45]. Protecting migrant children from vaccine preventable diseases is also an important public health concern [3]. It is important to highlight the need for community health centers and community health stations to provide comprehensive services for migrants. Furthermore, primary care institutions should provide more comprehensive services than tertiary hospitals in dealing with the mounting challenge of preventing non-communicable diseases [46]. Consistent with previous research [47], our study showed that migrants with chronic diseases were more likely to visit tertiary hospitals than community health centers or community health stations when they were sick.

Consistent with findings of a study conducted in Southern China [29], mean score for community orientation was the lowest among all domains, indicating unsatisfactory community-based health services. Community-oriented primary care takes into account the health care needs of not only the patients and families but also residents in the community [38]. Though community health centers and community health stations scored significantly higher when compared with tertiary hospitals, scores relating to this domain barely met minimum expectations and should be further improved. Previous literature emphasized that a team-oriented approach and linkages with the community were essential for community-oriented primary care services [48]. The paucity of community orientation services appears to be a notable problem.

The results indicated that migrants who were young, had not been in Guangzhou for long, migrated by oneself and were not expecting to remain permanently in Guangzhou tended to return to their hometown for primary care in township health centers or rural health stations when they were sick. In our study, more than half of the participants who accessed primary care in township health centers or rural health stations reported that insurance could cover parts of their health payment. A very significant reason for their home-returning health seeking behaviors can be attributed to their relatively special health insurance status [49]. By law, the rural-to-urban migrants should enroll in the New Rural Cooperative Medical Scheme (NRCMS), a rural health insurance scheme which applied to the healthcare received in the places of registration [50]. Migrants had to go back to their hometown for medical services if they want to use NRCMS since the NRCMS could not be used at temporary residences [49]. Also, massive disparity between cost of care in rural and urban areas for equivalent treatments forced migrants to return to their hometowns for treatment [25].

Factors positively associated with primary care experiences among migrants also included how migrants paid for their healthcare, as demonstrated by a previous study [36]. Cost may be a major deterrent for lower-income patients when accessing primary care [51]. In our study, migrants who reported their insurance covering parts of the medical expenses reported higher quality of primary care. In rural areas of China, people are covered by the NRCMS. As mentioned before, migrants would enroll in the NRCMS in the places of registration. In urban areas, people are covered by the Urban Resident Basic Medical Insurance (URBMI), which is for children, elderly and the unemployed, and Urban Employee Basic Medical Insurance (UEBMI), which is for the employed. The employed rural-to-urban migrants can have UEBMI provided by their employers at the residence. However, the employers are not required to provide UEBMI for migrants [49]. In our study, 85.2% of the participants reported having health insurance, whereas only 36.3% reported their insurance covering parts of the medical expenses. Migrants who reported insurance covering parts of the health payment might include those covered by NRCMS and went back to hometown for medical services, and those covered by UEBMI and had medical services at the residence. The plight of those without insurance covering the medical expenses should be a major policy concern. Inconsistent with other studies [28, 29], a self-reported good health status, having a chronic disease status and presenting of health insurances were not associated with better primary care quality. The PCAT total scores were independent of socio-demographic and migrant characteristics in the study. Further research is needed to elucidate the provider- and facility-related factors influencing the experiences of primary care.

Several limitations were identified in the study. First, some unmeasured confounders could mediate the relationship between the types of medical institutions and PCAT scores. Studies showed that practice organizational factors were associated with quality of primary care [52]. As our data were not gathered from the selected medical institutions, provider factors and facility factors could not be captured. Based on the literature review [14, 23, 29, 36, 53], potential patient factors were taken into account in this study. Second, we used the question “Is there a doctor or place that you usually go if you are sick or need advice about your health?” to define the medical institution for primary care. Migrants who reported municipal hospital or tertiary hospital as their source of primary care might consult frequently for specific health problems more than primary care. However, it could not distort the conclusions because these participants would also have experiences with primary care in the reported municipal hospital or tertiary hospital. Third, the sampling of the migrants was not randomized. We approached the participants in each selected workplace or community until the sample size was met. Nevertheless, we stipulated the distribution of employment status of the samples, with the purpose of ensuring the samples could be representative of the migrant population. We have also compared the characteristics between the migrant population of Guangzhou and the participants of our survey. Fourth, a cross-sectional survey did not allow us to explore causality from these findings. In our study, the phone numbers of the participants were recorded, thus a follow-up study could be a next important step. Future study could use a longitudinal approach to record patients’ primary care experiences over time and examine the impact of type of setting as well as other factors that are associated with improvement in quality and the care experience. Fifth, the survey was based on participant self-report, so recall bias was possible. However, this data collection method was necessary because our study focused on the migrants’ access to primary care and their perceived challenges after migrating to Guangzhou, which could not be shown with other methods [26]. Finally, the inferences drawn from the study may not apply to migrants in general but apply to migrants who access primary care under these types of medical institutions. However, we provided the overall picture of migrants’ primary care experiences, not just the frequent visitors. Despite these limitations, to our knowledge, this study is the first in China on migrants’ primary care quality and has significant implication for healthcare policy in China.

## Conclusions

This study demonstrates that migrants receiving primary care from township health centers, rural health stations, community health centers and community health stations reported worse primary care experiences than those receiving primary care from tertiary hospitals. There is much room for improvement in primary care quality provided by primary care institutions for rural-to-urban migrants. Relevant policies of medical insurance should be implemented to better provide affordable healthcare services to migrants accessing primary care. Primary care institutions performed poorly, further research exploring the specific reasons for poorer PCAT scores of community health center and community health station users will be needed.

## Supporting Information

S1 Appendix Primary Care Assessment Tool—Adult Short Version. (DOC)
