# Supplementary material for: Rural-to-Urban Migrants' Experiences with Primary Care under Different Types of Medical Institutions in Guangzhou, China
Source: PLoS One. 2015 Oct 16;10(10):e0140922. doi: 10.1371/journal.pone.0140922 (PMC4608723; doi:10.1371/journal.pone.0140922)
Supplement: S1 Appendix — (DOC) [file pone.0140922.s001.doc]

**PRIMARY CARE ASSESSMENT TOOL – ADULT SHORT VERSION**

| B. FIRST CONTACT - UTILIZATION | | | | | | |
| --- | --- | --- | --- | --- | --- | --- |
| Please check the one best answer. | | Definitely | Probably | Probably not | Definitely not | Not  sure/don’t remember |
| B1. | When you need a regular general checkup, do you go to your PCP before going somewhere else? | 4□ | 3□ | 2□ | 1□ | 9□ |
| B2. | When you have a new health problem, do you go to your PCP before going somewhere else? | 4□ | 3□ | 2□ | 1□ | 9□ |
| B3. | When you have to see a specialist, does your PCP have to approve or give you a referral? | 4□ | 3□ | 2□ | 1□ | 9□ |
|  | | | | | | |
| C. FIRST CONTACT - ACCESS | | | | | | |
| Please check the one best answer. | | Definitely | Probably | Probably not | Definitely not | Not  sure/don’t remember |
| C3. | When your PCP is *open* and you get sick, would someone from there see you the same day? | 4□ | 3□ | 2□ | 1□ | 9□ |
| C4. | When your PCP is *open*, can you get advice quickly over the phone if you need it? | 4□ | 3□ | 2□ | 1□ | 9□ |
| C5. | When your PCP is *closed*, is there a phone number you can call when you get sick? | 4□ | 3□ | 2□ | 1□ | 9□ |
| C7. | When your office is closed during the night and patients get sick, would someone from your office be able to see them that night? | 4□ | 3□ | 2□ | 1□ | 9□ |

| D. ONGOING CARE | | | | | | |
| --- | --- | --- | --- | --- | --- | --- |
| Please check the one best answer. | | Definitely | Probably | Probably not | Definitely not | Not  sure/don’t remember |
| D1. | When you go to your PCP’s, are you taken care of by the *same* doctor or nurse each time? | 4□ | 3□ | 2□ | 1□ | 9□ |
| D4. | If you have a question, can you call and talk to *the doctor or nurse who knows you best*? | 4□ | 3□ | 2□ | 1□ | 9□ |
| D7. | Does your PCP know you very well as a *person*, rather than as someone with a medical problem? | 4□ | 3□ | 2□ | 1□ | 9□ |
| D9. | Does your PCP know what problems are most important to you? | 4□ | 3□ | 2□ | 1□ | 9□ |
|  | | | | | | |
| E. COORDINATION (REFERRALS) | | | | | | |
| E2. | Have you ever had a visit to any kind of specialist or special service? | | | | | |
|  | 1□ Yes 2□ No (**Skip to question F1**) 9□ Not sure/don’t remember (**Skip to question F1**) | | | | | |
| Please check the one best answer. | | Definitely | Probably | Probably not | Definitely not | Not  sure/don’t remember |
| E8. | Did your PCP discuss with you different places you could have gone to get help with that problem? | 4□ | 3□ | 2□ | 1□ | 9□ |
| E9. | Did your PCP or someone working with your PCP help you make the appointment for that visit? | 4□ | 3□ | 2□ | 1□ | 9□ |
| E10. | Did your PCP write down any information for the specialist about the reason for the visit? | 4□ | 3□ | 2□ | 1□ | 9□ |
| E12. | After you went to the specialist or special service, did your PCP talk with you about what happened at the visit? | 4□ | 3□ | 2□ | 1□ | 9□ |

| F. COORDINATION (INFORMATION SYSTEMS) | | | | | | |
| --- | --- | --- | --- | --- | --- | --- |
| Please check the one best answer. | | Definitely | Probably | Probably not | Definitely not | Not  sure/don’t remember |
| F1. | When you go to your PCP, do you bring any of your own medical records, such as shot records or reports of medical care you had in the past? | 4□ | 3□ | 2□ | 1□ | 9□ |
| F2. | Could you look at your medical record if you wanted to? | 4□ | 3□ | 2□ | 1□ | 9□ |
| F3. | When you go to your PCP, is your medical record always available? | 4□ | 3□ | 2□ | 1□ | 9□ |
|  | | | | | | |
| G. COMPREHENSIVENESS (SERVICES AVAILABLE) | | | | | | |
| Please check the one best answer. | | Definitely | Probably | Probably not | Definitely not | Not  sure/don’t remember |
| Following is a list of services that you or your family might need at some time. For each one, please indicate whether it is available at your PCP’s office. | |  |  |  |  |  |
| G2. | Immunizations (shots) | 4□ | 3□ | 2□ | 1□ | 9□ |
| G6. | Family planning or birth control methods | 4□ | 3□ | 2□ | 1□ | 9□ |
| G8. | Counseling for mental health problems | 4□ | 3□ | 2□ | 1□ | 9□ |
| G10. | Sewing up a cut that needs stitches | 4□ | 3□ | 2□ | 1□ | 9□ |

| H. COMPREHENSIVENESS (SERVICE PROVIDED) | | | | | | |
| --- | --- | --- | --- | --- | --- | --- |
| The next questions deal with different types of health care services that you sometimes get. Please check the one best answer. | | | | | | |
|  | | Definitely | Probably | Probably not | Definitely not | Not  sure/don’t remember |
| In visits to your PCP, are any of the following subjects discussed with you? | |  |  |  |  |  |
| H1. | Advice about healthy foods and unhealthy foods or getting enough sleep | 4□ | 3□ | 2□ | 1□ | 9□ |
| H2. | Home safety, like getting and checking smoke detectors and storing medicines safely | 4□ | 3□ | 2□ | 1□ | 9□ |
| H4. | Ways to handle family conflicts that may arise from time to time | 4□ | 3□ | 2□ | 1□ | 9□ |
| H5. | Advice about appropriate exercise for you | 4□ | 3□ | 2□ | 1□ | 9□ |
| H7. | Checking on and discussing the medications you are taking | 4□ | 3□ | 2□ | 1□ | 9□ |
|  | | | | | | |
| I. FAMILY - CENTEREDNESS | | | | | | |
| These next questions are about the relationship of your health care provides with your family. Please check the one best answer. | | | | | | |
|  | | Definitely | Probably | Probably not | Definitely not | Not  sure/don’t remember |
| I1. | Does your PCP ask you about *your* ideas and opinions when planning treatment and care for you or a family member? | 4□ | 3□ | 2□ | 1□ | 9□ |
| I2. | Has your PCP asked about illnesses or problems that might run in your family? | 4□ | 3□ | 2□ | 1□ | 9□ |
| I3. | Would your PCP meet with members of your family if you thought it would be helpful? | 4□ | 3□ | 2□ | 1□ | 9□ |
|  | | | | | | |
| J. COMMUNITY ORIENTATION | | | | | | |
| Please check the one best answer. | | Definitely | Probably | Probably not | Definitely not | Not  sure/don’t remember |
| J1. | Does anyone at your PCP’s office ever make home visits? | 4□ | 3□ | 2□ | 1□ | 9□ |
| J2. | Does your PCP know about the important health problems of your neighborhood? | 4□ | 3□ | 2□ | 1□ | 9□ |
| J3. | Does your PCP get opinions and ideas from people that will help to provide better health care? | 4□ | 3□ | 2□ | 1□ | 9□ |

| K. CULTURALLY COMPETENT | | | | | | |
| --- | --- | --- | --- | --- | --- | --- |
| Please check the one best answer. | | Definitely | Probably | Probably not | Definitely not | Not  sure/don’t remember |
| K1. | Would you recommend your PCP to a friend or relative? | 4□ | 3□ | 2□ | 1□ | 9□ |
| K2. | Would you recommend your PCP to someone who does not speak English well? | 4□ | 3□ | 2□ | 1□ | 9□ |
| K3. | Would you recommend your PCP to someone who uses folk medicine, such as herbs or homemade medicines, or has special beliefs about health care? | 4□ | 3□ | 2□ | 1□ | 9□ |
